# An assessment of the vaccination of school-aged children in England against SARS-CoV-2

**DOI:** 10.1186/s12916-022-02379-0

**Published:** 2022-05-18

**Authors:** Matt J. Keeling, Sam E. Moore

**Affiliations:** The Zeeman Institute for Systems Biology & Infectious Disease Epidemiology Research, School of Life Sciences and Mathematics Institute, University of Warwick, Coventry, CV4 7AL UK

**Keywords:** COVID-19, Immunisation, Infection, Hospital admission

## Abstract

**Background:**

Children and young persons are known to have a high number of close interactions, often within the school environment, which can facilitate rapid spread of infection; yet for SARS-CoV-2, it is the elderly and vulnerable that suffer the greatest health burden. Vaccination, initially targeting the elderly and vulnerable before later expanding to the entire adult population, has been transformative in the control of SARS-CoV-2 in England. However, early concerns over adverse events and the lower risk associated with infection in younger individuals means that the expansion of the vaccine programme to those under 18 years of age needs to be rigorously and quantitatively assessed.

**Methods:**

Here, using a bespoke mathematical model matched to case and hospital data for England, we consider the potential impact of vaccinating 12–17 and 5–11-year-olds. This analysis is reported from an early model (generated in June 2021) that formed part of the evidence base for the decisions in England, and a later model (from November 2021) that benefits from a richer understanding of vaccine efficacy, greater knowledge of the Delta variant wave and uses data on the rate of vaccine administration. For both models, we consider the population wide impact of childhood vaccination as well as the specific impact on the age groups targeted for vaccination.

**Results:**

Projections from June suggested that an expansion of the vaccine programme to those 12–17 years old could generate substantial reductions in infection, hospital admission and deaths in the entire population, depending on population behaviour following the relaxation of control measures. The benefits within the 12–17-year-old cohort were less marked, saving between 660 and 1100 (95% PI (prediction interval) 280–2300) hospital admissions and between 22 and 38 (95% PI 9–91) deaths depending on assumed population behaviour. For the more recent model, the benefits within this age group are reduced, saving on average 630 (95% PI 300–1300) hospital admissions and 11 (95% PI 5–28) deaths for 80% vaccine uptake, while the benefits to the wider population represent a reduction of 8–10% in hospital admissions and deaths. The vaccination of 5–11-year-olds is projected to have a far smaller impact, in part due to the later roll-out of vaccines to this age group.

**Conclusions:**

Vaccination of 12–170-year-olds and 5–11-year-olds is projected to generate a reduction in infection, hospital admission and deaths for both the age groups involved and the population in general. For any decision involving childhood vaccination, these benefits needs to be balanced against potential adverse events from the vaccine, the operational constraints on delivery and the potential for diverting resources from other public health campaigns.

**Supplementary Information:**

The online version contains supplementary material available at (10.1186/s12916-022-02379-0).

## Background

The SARS-CoV-2 pandemic has lead to severe healthcare burdens, often necessitating unprecedented non-pharmaceutical intervention measures [1–4]. From December 2020, the availability of highly effective vaccines (first the Pfizer-BioNTech, followed by the Oxford-AstraZeneca and Moderna vaccines) offered an alternative method for limiting infection and disease [5–7]. In England, this led to the largest ever vaccine campaign with around 100 million doses administered in the first twelve months. These vaccines were offered in a targeted manner, with healthcare workers, the vulnerable and the elderly being the first to be offered their initial dose [8].

One of the key characteristics of the SARS-CoV-2 pandemic has been the striking age-dependent nature of disease severity, with older (and clinically vulnerable) individuals suffering a far higher burden of hospital admissions and deaths [9–12]. Many countries initially closed schools as a response to this pandemic [13–16], but the role played by school-aged children and the strength of transmission within the school environment remains deeply contested with different conclusions being drawn from a range of modelling and data-driven analyses [17–33]. This dichotomy of views on the role of schools may be attributed to two conflicting factors. Firstly, all the available data highlights that younger individuals have a lower susceptibility to SARS-CoV-2 infection, a reduced risk of displaying symptoms and a greatly reduced risk of severe illness [9, 11, 33, 34]. However, it is also well established that the school environment is historically associated with a high transmission risk for many respiratory pathogens [35–39], simply due to the large number of pupils and the high number of contacts involved [40–42]. The data from England suggests that (before the emergence of the Omicron variant) there had not been the type of explosive outbreaks of SARS-CoV-2 within schools that are frequently associated with other respiratory diseases [43–46]; yet it is evident that transmission does occur within schools [47–49] and 2021 saw pronounced increases in school-aged infection in England [45, 50].

Here a complex age-structured model, matched to data in the seven NHS (National Health Service) regions of England [51–53], is used to examine the vaccination of 12–17 and 5–11-year-olds. In particular, the number of infections, hospital admissions and deaths in both the whole population and the target age groups are compared with and without vaccination. The model has been extensively used throughout the pandemic, particularly to answer questions about vaccination [53, 54] and to project the likely impact of relaxing control measures [55, 56]. This model formulation has also been used to consider the impact of school reopening on the population level incidence [17], although bespoke individual-scale models may be more appropriate when focusing on within-school dynamics rather than their feed-back on the community [26, 57, 58].

We consider the projected dynamics and the benefits from the perspective of a pre-Omicron assessment. The rapid spread of the Omicron variant has dramatically shifted the epidemiological landscape with far more infections expected in England during very early 2022. However, there are currently too many uncertainties surrounding fundamental parameters associated with this new variant to make robust quantitative projections.

The aim of this paper is not to argue for or against vaccination of particular age groups, but to present a quantitative assessment of the epidemiological role that vaccination has played and is likely to play in the near future. As such, the paper focuses on the positives of vaccination: the impact of vaccinating children and young people on the infection in both the vaccinated age group in particular and the population in general. There is no quantitative consideration or comparison of these benefits against potential risks or costs.

## Methods

Three sets of simulations are performed: one from the 26th June 2021 which was used as part of the JCVI (Joint Committee on Vaccination and Immunisation) decision-making process for the vaccination of 12–17-year-olds; one using additional data and information up to 6th November 2021, again looking at the benefits of vaccinating 12–17-year-olds; and a final simulation, also based on 6th November data, examining the benefit of vaccinating 5–11-year-olds. These two models use the data that was available on 26th June and 6th November to infer parameter values and generate projections until the end of 2022. The models were the basis of the July 2021 and October 2021 Roadmap documents respectively [59, 60], which considered the likely dynamics of COVID-19 infections and hospital admissions as control measures were relaxed; analysis has shown that these Roadmap projections generated a reliable estimation of medium-term trends [61].

The model used throughout this paper is an age-structured formulation that has been developed throughout this pandemic [17, 51, 56] and has been continually matched to a range of epidemiological data [52]. The model is explained in more detail in the Additional file 1, but a brief overview of the main characteristics are given here. The model is a deterministic age-structured model, partitioning the population into twenty-one 5-year age classes and spatially into the seven NHS regions of England. The model captures age-dependent mixing, susceptibility, likelihood of symptoms and likelihood of severe disease. It has been adapted throughout the course of the pandemic to include multiple variants [62] and vaccination [8, 53]. The November model also includes the waning of vaccine protection and infection-derived immunity as well as the deployment of booster vaccination. One of the principal drivers of epidemic behaviour is the mixing of individuals in the population, which can be modified either as a result of imposed restrictions or voluntary behaviour change due to perceived risk. This level of precautionary behaviour is inferred as a generally slowly varying parameter except immediately following changes to legislation, capturing the fact that at other times we do not expect very rapid changes in social mixing or other forms of precautionary measures. This and other parameters are inferred by matching to six key observations of the epidemic in each of the seven spatial regions [52]: the proportion of community tests that return a positive result, the number of daily hospital admissions and deaths, the number of hospital beds and intensive care unit beds that are occupied by COVID patients, and the proportion of S-gene targeted tests (TaqPath PCR tests) that are positive—with the latter providing a proxy measure for the transition between major variants [63, 64]. The results we present need to account for the parameter variability inherent in our Bayesian fitting methodology [52]; alongside means we therefore report 95% prediction intervals such that 95% of simulations, using the posterior parameter distributions, lie within the interval. When comparing different vaccination strategies, the impact of the vaccine strategy is calculated for each set of parameters, and then the distribution of the impact across all parameters is assessed.

The model is used to investigate the number of infections, hospital admissions and deaths using a weighted sum over a given time frame (we define *w*_*d*_ as the weighting applied on day *d*). Numbers with and without the vaccination of children are compared, considering both the total numbers across the entire population and numbers in the cohort being vaccinated. The results generated in June 2021, and presented to JCVI, used a time window of 19th July 2021 until 31st December 2021 and weighted all time-points equally (Additional file 2) - with 19th July chosen as the start of Step 4 and the lifting of restrictions. However, it is arguably more appropriate to consider a longer time window (19th July 2021 until 31st December 2022) so that the longer impact of immunisation can be captured. We do this by having a constant weighting applied from 19th July to 31st December 2021, as previously, followed by weights that decrease linearly to zero by 31st December 2022. This gradual decline accounts for the greater uncertainty in longer-term projections and has the advantage over a discrete step-change that the results are relatively insensitive to the timing of future waves of infection. In the earlier step-change formulation a wave of infection in December 2021 would be included in the sum, but one in January 2022 would not; the use of a longer window with a gradual decline in the weighting overcomes many of these problems. The use of such a weighted time window draws into sharp focus the need to specify the total time frame over which any vaccine programme is evaluated; we quantify this by calculating the programme weighting, *T*_*V*_, which is the total weighted time over which the vaccination of the target cohort operates:

$$T_{V} = \frac{ \sum_{d} V_{d} \sum_{D>d} w_{D} }{\sum_{d} V_{d}}, $$

where *V*_*d*_ is the number of individuals in the cohort vaccinated on day *d*.

One potential difficulty with the modelling methodology is the difference in age groups used within the different data streams. The model uses regular 5-year age groups (0–4, 5–9, etc.), based on the 5-year age structure of the contact matrices [41]; hospital data is aggregated into irregular partitions for younger age groups (0–5, 6–17, 18–24); while vaccine cohorts (over 18s, 16–17, 12–15 and 5–11) are based on a range of medical considerations. One potential solution would be to use yearly age classes (as in [17]), but this increases computational overheads and requires strong assumptions to be made about age structure of the contacts especially within year groups. Here, we make the a simpler assumption that the dynamics are homogeneous within a 5-year age group and there are equal numbers within each year group; as such the number of 0–5-year-olds admitted to hospital is calculated as 100% of the 0–4 age group plus 20% of the 5–9 age group. Similarly, if vaccinating 80% the aged 12–15 cohort, we model vaccinating 48% (60% × 80%) of the 10–14 age group and 16% (20% × 80%) of the 15–19 age group.

### June projections

**Fig. 1:**
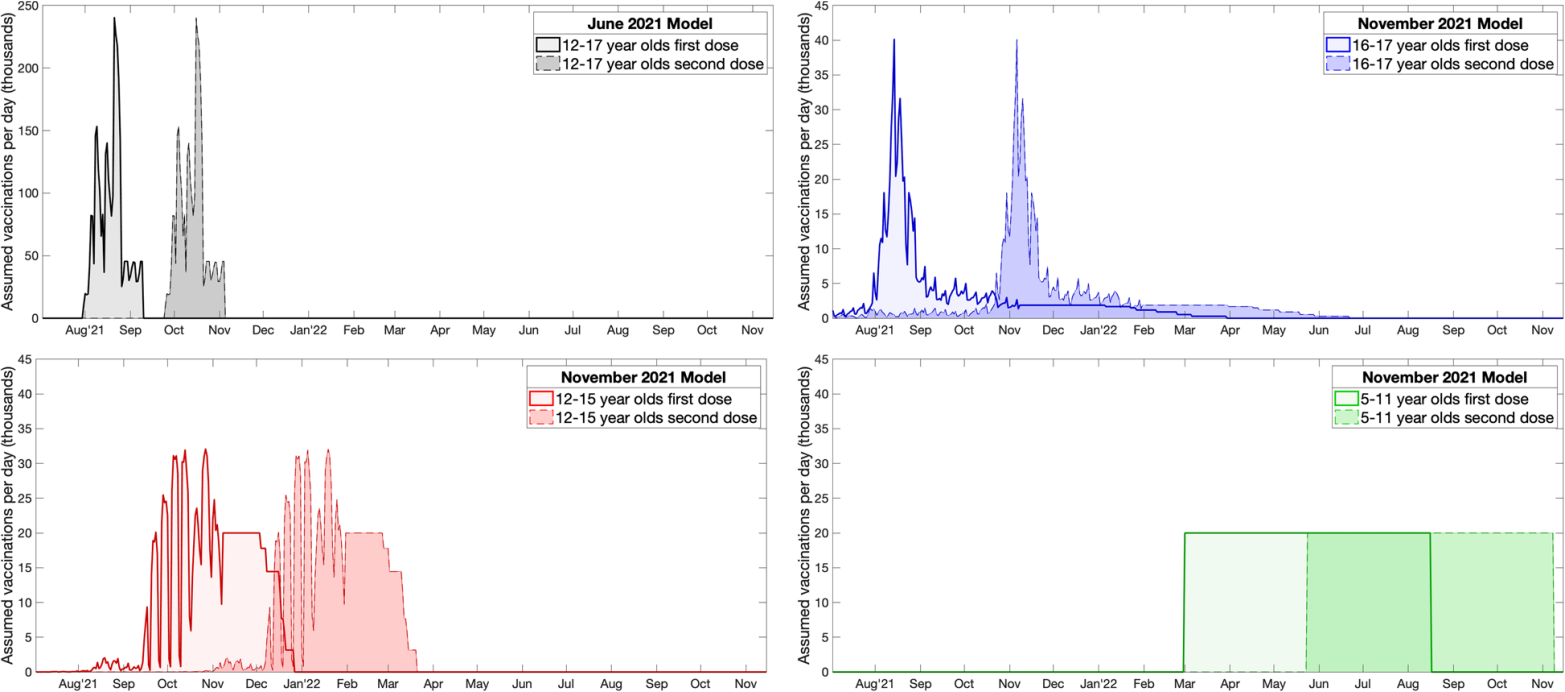
Timing of vaccine delivery in the two models (June: black, November: blue, red, green) by target age group. Solid outlines and paler shading correspond to first doses, while dashed outlines and darker shading correspond to second doses. For the June model the vaccination of 12–17-year-olds is assumed to be part of the national immunisation campaign. For the November model, first doses are largely based on reported numbers until early November; the vaccination of 12–15-year-olds and 5–11-year-olds is from the default assumption of 140,000 vaccines per week through the school delivery programme, and a 70% uptake

The model from the 26th June was based on the model framework used for the 6th July Roadmap [56]. Vaccination of 12–17-year-olds was included as part of the national vaccine roll-out, which was assumed to administer 2 million doses a week from late July onward, with 12–17-year-olds to be offered vaccination once older groups had been received theirs. Throughout it was also assumed that 80% of this age group would take the vaccine. Given the need to first vaccinate, those over 18 years old and to offer second doses at the correct time, the vast majority of first doses in the 12–17 age group take place between early August and late September 2021. It was assumed that second doses are given no sooner that 8 weeks after the first dose, with precise timing dependant on the vaccine schedule leading to the majority of second doses in 12–17-year-olds being administered between early October and late November 2021 (Fig. 1).

In late June 2021, the vaccine efficacy against the newly emerged Delta variant remained highly uncertain, with the best estimates suggesting limited impact from the first dose but markedly increased protection from the second dose. Although 12–17-year-olds were largely given the Pfizer vaccine, Table 1 presents vaccine efficacy values from the time for both AstraZeneca and Pfizer as the protection from AstraZeneca is important for the rest of the population.

**Table 1:** Estimated vaccine efficacy (from the June projections) against infection, symptomatic disease, hospital admission and death after one or two doses with either the AstraZeneca ChAdOx vaccine or an mRNA vaccine such as the Pfizer vaccine. Values were taken from PHE reports at the time

Protection against:	AstraZeneca		Pfizer	
	Dose 1	Dose 2	Dose 1	Dose 2
Infection	34%	71%	34%	73%
Symptoms	34%	82%	34%	83%
Hospitalisation	64%	90%	64%	91%
Deaths	60%	96%	60%	96%

These June simulations were performed before ‘Step 4’ (on 19th July 2021) when all restrictions on social mixing were relaxed, therefore a range of future possibilities were explored. This relaxation of precautionary behaviour is modelled as a step-change on 19th July 2021 (of four different sizes), followed by a gradual return to pre-pandemic mixing with different assumptions achieving normality between August 2021 and February 2022. This uncertainty in population behaviour, and hence transmission, determines the scale and shape of the Delta variant wave, with more rapid returns to pre-pandemic mixing leading to higher but shorter waves of infection.

Simulation results are aggregated either across the short-term time window (19th July–31st December 2021, with equal weighting on all days) or the longer-term window (with constant weighting from 19th July–31st December 2021, and then a linearly declining weighting until 31st December 2022). For vaccinations that begin in early August, this means that the short-term window has a programme weighting of 134 days over which childhood vaccination can impact the dynamics, which extends to around 316 days for the longer-term window.

### November Projections

The more recent model uses data and parameters up to 6th November to perform similar analyses, this data includes updated vaccine efficacy estimates (Table 2) which provide greater protection especially after the first dose compared to the earlier estimates. However, by this point in time, data were also accumulating on the efficacy of vaccines over time, with a strong signal of waning protection. Given this observation, from September 2021 booster (third) doses were offered to individuals who had received their second dose of vaccine more than 6 months ago. Both waning and boosters are included in the updated model framework [54].

**Table 2:** Estimated vaccine efficacy from the November models against infection, symptomatic disease, hospital admission and death after one or two doses with either the AstraZeneca ChAdOx vaccine or an mRNA vaccine such as the Pfizer vaccine. Values are taken from UKHSA reports [65, 66]

Protection against:	AstraZeneca		Pfizer	
	Dose 1	Dose 2	Dose 1	Dose 2
Infection	45%	70%	55%	85%
Symptoms	45%	70%	55%	90%
Hospitalisation	80%	95%	80%	95%
Deaths	80%	98%	80%	98%

Given that the uncertainties in behaviour associated with the transition to Step 4 had been largely resolved, the November model focuses on a range of other unknowns including the speed of vaccine delivery and the level of uptake. The level of population mixing is determined by extrapolation of the general pattern of increased mixing that was seen from February to November 2021, such that mixing would return to pre-pandemic levels by early 2022. By this point, vaccination of 12–15-year-olds was taking place in the school environment, separating it from the logistical demands of vaccinating the rest of the population, while first doses had been offered to 16–17-year-olds since August 2021. It is generally considered more equitable to vaccinate children though a school-based system, which also generally achieves better up-take than other methods. In these simulations the counterfactual scenario in which 12–17-year-olds are not vaccinated is first considered; this is compared to projections that use the observed vaccinations until 6th November (by which time 70% of 16–17-year-olds and 40% of 12–15-year-olds have received one dose) and then continues with vaccination of 12–15-year-olds into the future until the required uptake (60%, 70% or 80%) is achieved (Fig. 1). Sensitivity to the rate at which the vaccine roll-out continues from 6th November onwards is explored, vaccinating either 100,000, 140,000 or 180,000 12–15 year old pupils per week. For simplicity it is assumed that a second dose is given 12 weeks after the first (Fig. 1).

For these November simulations only the longer-term window is used (with constant weighting from 19th July to 31st December 2021, and then a linearly declining weighting until 31st December 2022). Given that vaccination of 12–15 -year-olds began on 20th September 2021 and continued throughout 2021, this means that childhood vaccination has a programme weighting of 267 days.

This more recent model is also used to examine the vaccination of 5–11-year-olds, assuming that this begins in March 2022 once second doses of 12–15 years is largely complete—logistical constraints make it unlikely that school-based programmes for 12–15-year-olds and 5–11-year-olds could operate in parallel. Vaccination of this age group is assumed to be through the school environment and a range of vaccine uptake levels (60%, 70% and 80%) and a range of deployment speeds (slow - 100,000 per week, default - 140,000 per week or fast - 180,000 per week) are considered as part of the sensitivity analysis (Fig. 1). Given that there are approximately 87% more 5–11-year-olds than 12–15-year-olds in England, vaccination of this cohort is a far lengthier process. The longer-term window is again used, which given that vaccination does not begin until March 2022, means that vaccination of 5–11-year-olds has a programme weighting of only 81 days.

## Results

**Fig. 2:**
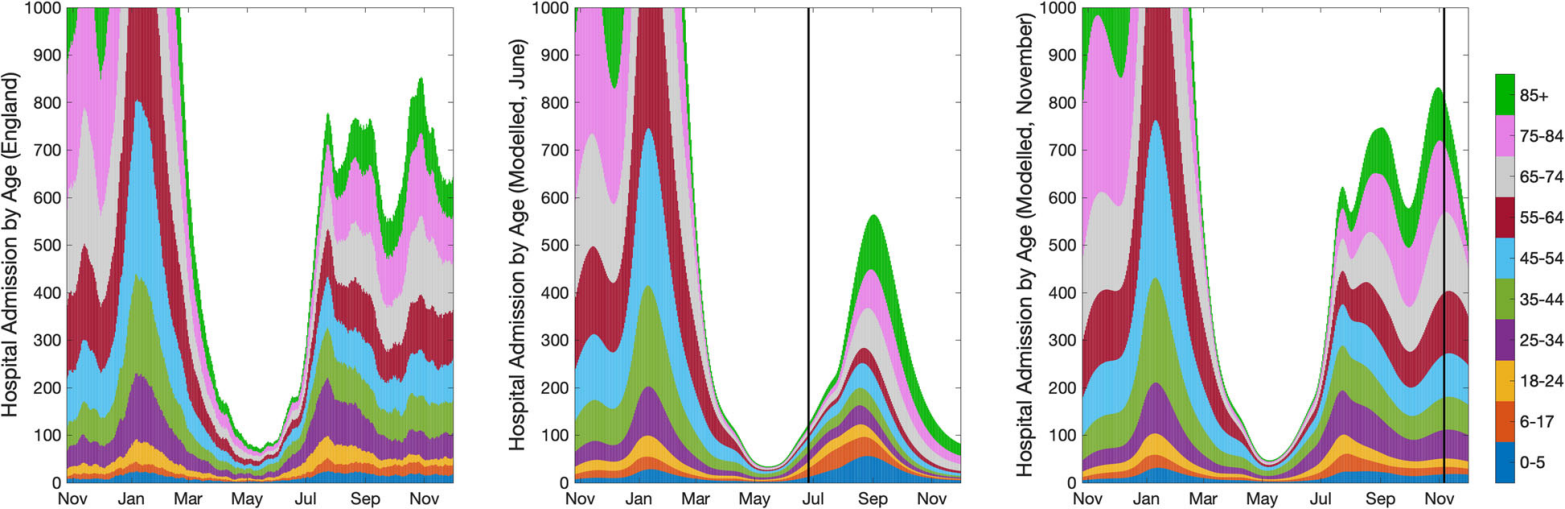
Comparison of data (left) and model results (June: centre, November: right) for the number of daily hospital admissions in ten age groups, from 0–5 years to 85 and above. The data on the left is a 1-week moving average (using 3 days either side of the date) which removes any day-of-the-week effects and helps to smooth stochastic fluctuations. The model results are from the both June (average over seven Step 4 mixing scenarios) and November predictions and are computed using 5-year age classes, and as such the first three age groups shown are composites of multiple simulated age classes: the 0–5 age group contains all projected hospital admissions from the 0–4 age class and one fifth of all admissions from the 5–9 age-class. The vertical lines corresponds to the simulation dates for the two projections—26th June and 6th November 2021

We begin by comparing age-structured model output (from the June and November models) against the data for the number of individuals admitted to hospital in ten different age groups, from 0–5 to over 85 years old (Fig. 2). The June results (which are an average over the seven Step 4 scenarios investigated [56], including vaccination of 12–17-year-olds) highlight an underestimation of the epidemic scale, but an overestimation of young people being hospitalised—this was principally due to the inability to accurately forecast population level mixing in response to the Step 4 relaxation step. For the November simulations, there is a much closer agreement between model results and the data. These later replicates were performed by fitting to the total hospital admissions (without age structure) over this period, so have been matched to the historic changes in imposed restrictions and precautionary mixing that drive the dynamics. Nevertheless, this comparison acts as a relatively independent assessment of the model fit, as age-structured data is not used as part of the inference process.

### June predictions

**Fig. 3:**
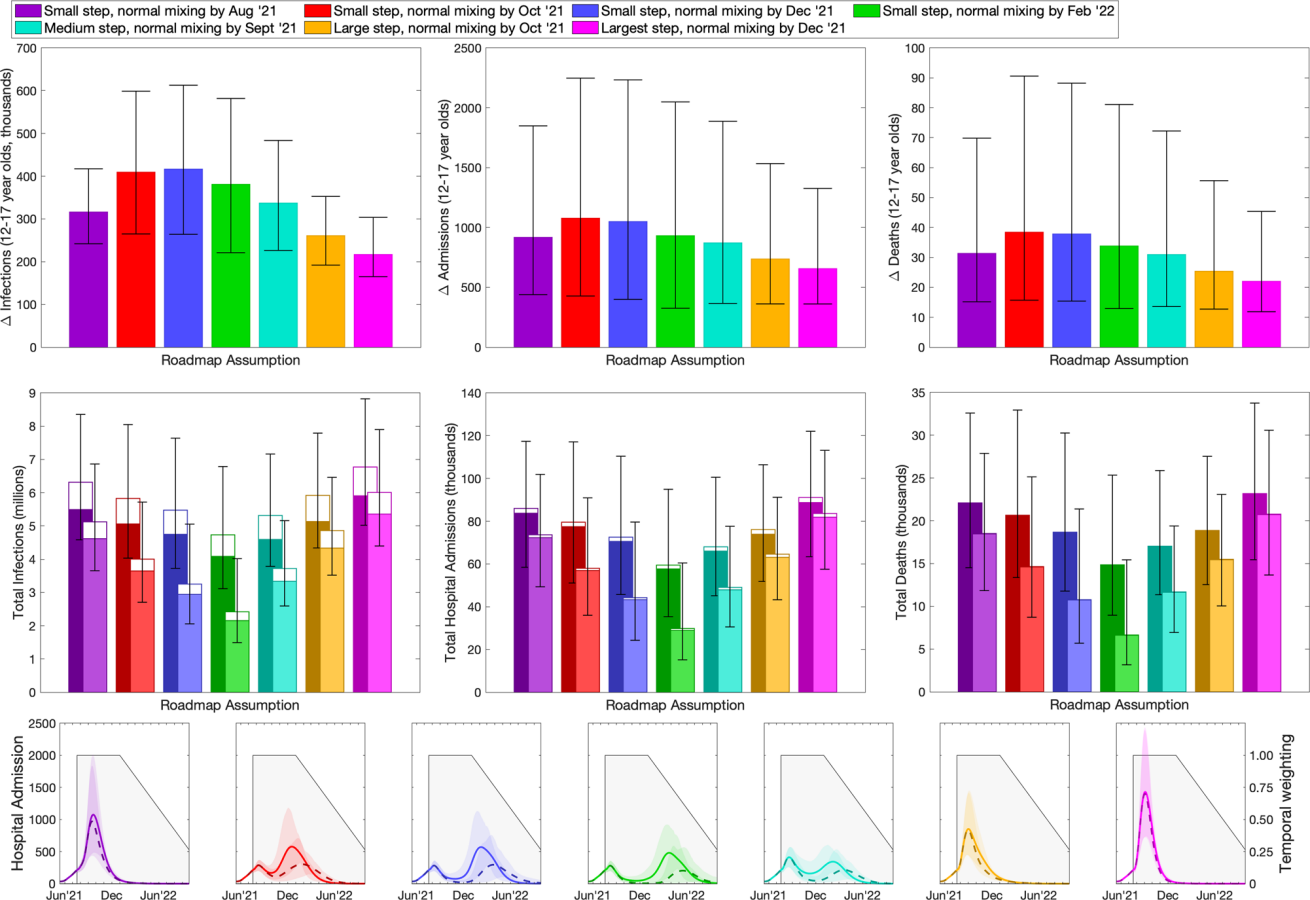
Impact of vaccination of 12–17-year-olds in England, calculated in June 2021. Top row: reduction in infections, hospital admissions and deaths in 12–17-year-olds due to vaccination in this age group. Middle row: total number of infections, hospital admissions and deaths in the entire population (total bar) and 12–17-year-olds (open bar)—the darker rear bars are the projected totals without vaccination of 12–17-year-olds, while the lighter front bars are the totals when 80% of 12–17-year-olds receive the vaccine. Lower row: number of projected hospital admissions over time (lines and ribbons), and the assumed time discounting (grey shading). In the top two rows bars are the mean value, error bars are the 95% prediction intervals. The different colours represent different assumptions about changes in social mixing patterns after Step 4 [56]; this is modelled as a step-change on 19th July 2021, followed by a gradual return to pre-pandemic mixing over different time scales

The projections generated in June 2021 had to make assumptions about the population mixing that would occur following Step 4 of the relaxation roadmap when all legal restrictions on social mixing were removed. A range of scenarios were investigated (bottom row, Fig. 3), from an abrupt return to pre-pandemic mixing (shown in purple and pink) to a far more gradual return (shown in green and cyan). Unsurprisingly, a rapid return to pre-pandemic mixing leads to a single sharp peak in infection and hospital admissions, whereas a more gradual return often leads to a multi-peaked wave. These plots of the total number of hospital admissions show results without (solid line) and with (dashed line) the vaccination of 12–17-year-olds.

The impact of childhood vaccination on the age classes concerned (top row, Fig. 3) leads to a reduction in the total number of infections (left), hospital admissions (centre) and deaths (right) that are projected over the period 19th July 2021 to 31st December 2022, with the appropriate temporal weighting. Similar results restricted to the period 19th July 2021 to 31st December 2021 are given in the Additional file 2. The assumption about relaxation to pre-pandemic behaviour (different colours in Fig. 3) has a large effect on the dynamics and hence the impact of vaccines on the 12–17-year-old age group. In general the results suggest that vaccination of 12–17 years (with around 3.8 million in this age-group in England) reduces infection in the same age group by 200–400 thousand, reduces hospital admissions by around 600–1100, and reduces death by around 20–40 individuals, although the 95% prediction intervals on each of these are large showing the considerable uncertainty in the dynamics. Compared to the counterfactual scenario, in which under 18s are not vaccinated, this equates to a mean 25–55% decrease in infection, hospital admissions and deaths in this age group, depending on the assumptions made about the return to pre-pandemic mixing.

Broadening our scope to the entire population of approximately 56 million people in England (middle row, Fig. 3) we consider the total number of infections, hospital admission and deaths over the time frame, both with (front lighter bars) and without (rear darker bars) vaccination of 12–17-year-olds. The top bar that is unfilled corresponds to the numbers in the 12–17 age group, hence the difference between the two unfilled bars is the value shown in the top row. Error bars refer to the uncertainty in the total values, given as 95% prediction intervals. As expected, the assumptions about mixing after Step 4 on 19th July 2021 have a substantial impact on the total numbers predicted and the impact of vaccinating 12–17-year-olds. In general, the total decrease in infection, hospital admissions and deaths is between 8% and 45% depending on the relaxation scenario. Slow relaxation to pre-pandemic mixing (e.g. green) leads to both lower totals and a greater impact of vaccination as the longer second (relaxation) wave generated when *R* just exceeds one is suppressed by vaccination. In contrast, for rapid relaxation (e.g. pink) there is insufficient time for the vaccination of 12–17 years to substantially reduce the infection dynamics. These graphs also highlight the lower severity of disease generally experienced by younger age groups; the fraction of each bar that is unfilled (representing those aged 12–17 years old) decreases substantially from infection, to hospital admission, to death.

### November predictions, 12–17-year-olds

**Fig. 4:**
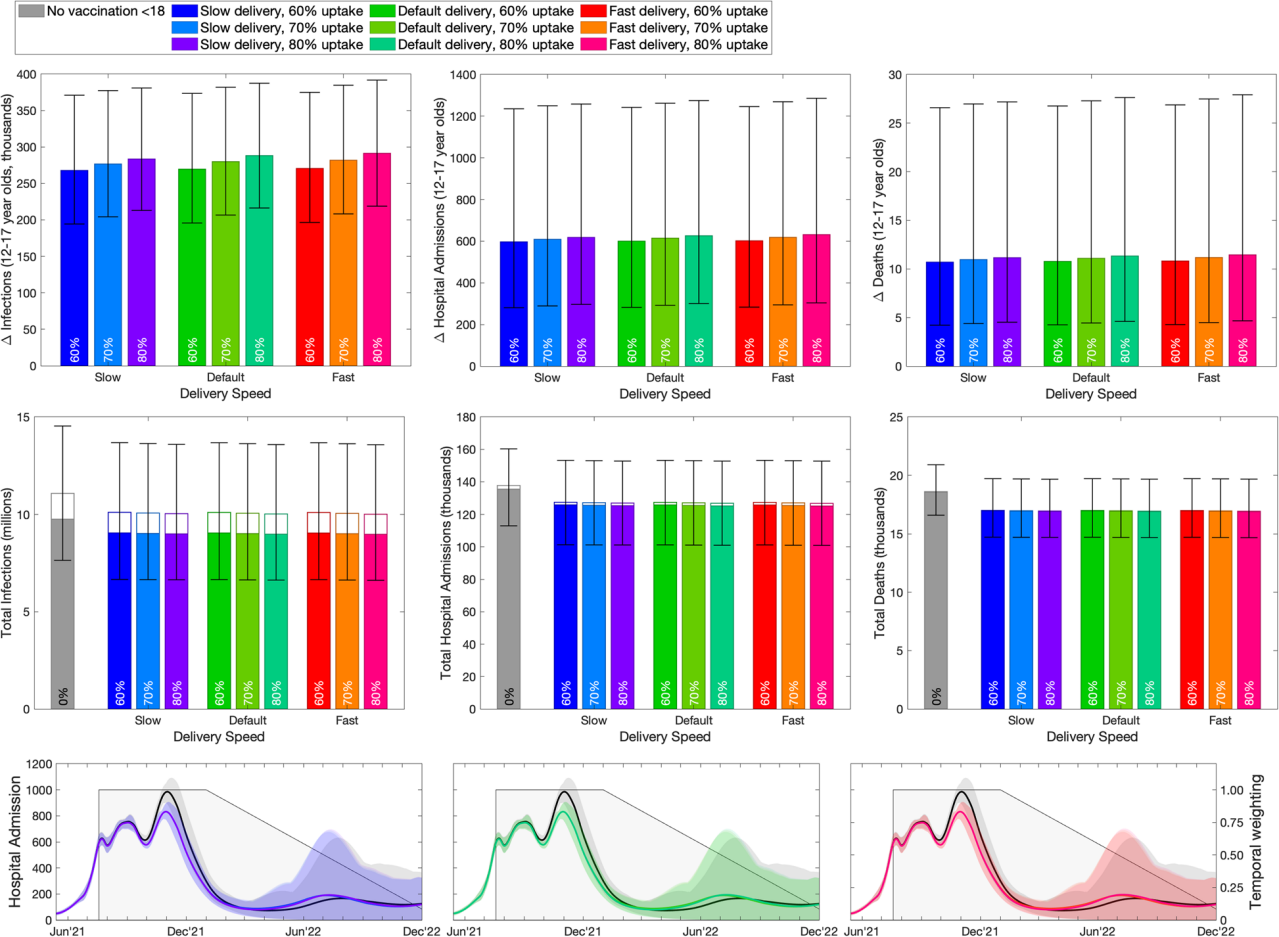
Impact of vaccination of 12–17-year-olds in England, calculated in November 2021. Top row: reduction in infections, hospital admissions and deaths in 12–17-year-olds due to vaccination in this age group. Middle row: total number of infections, hospital admissions and deaths in the entire population (total bar) and 12–17-year-olds (open bar). Lower row: number of projected hospital admissions over time (lines and ribbons; black for without childhood vaccination, colours corresponding to the bars above with 70% uptake and default speed), and the assumed time discounting (grey shading). In the top two rows bars are the mean value, error bars are the 95% prediction intervals, and different colours represent different assumptions about uptake in 12–15-year-olds (no vaccination, 60%, 70% and 80%) and different assumptions about vaccine delivery speed (slow - 100,000 per week, default - 140,000 per week, fast - 180,000 per week). In 16–17-year-olds, uptake was based on observations up to mid November except the counterfactual of no vaccination in which case all vaccination in 12–17-year-olds were removed

Rerunning similar analysis in November, with greater understanding of vaccine efficacy against the Delta variant and hindsight for the social mixing after Step 4, leads to broadly similar patterns but with slightly reduced benefits (Fig. 4). The lower panels again show examples of the total number of daily hospital admissions projected over time, together with the temporal weighting that is applied between 19th July 2021 and 31st December 2022. In these panels, the black curve (and associated 95% prediction interval) refers to the counterfactual epidemic in the absence of childhood vaccination, while the blue, green and red curves show the impact of childhood vaccination with a slow, default and fast roll-out from November onward, respectively.

Focusing on the implications for vaccination of 12–17-year-olds on this age group (top row, Fig. 4), we project an approximate saving of around 270–290 thousand infections, 600–630 hospital admission and 11 deaths—which corresponds to a mean 20–28% drop in these quantities compared to the total in this age group. Delivery speed (blue, green and red) has relatively limited impact on the dynamics - in part because the vaccination of 12–17-year-olds up to the 6th November 2021 is set by the observed schedule, which also restricts the timing of second doses. There is a more pronounced impact of vaccine uptake, with higher uptakes generating a greater impact. As seen with the earlier projections, there is a far greater reduction in the absolute number of infections compared to hospital admissions and deaths. Contrasting the June and November results, shows a lower benefit (approximately halved) in all three measures from the revised November model compared to the results from June. This is partly attributable to the larger wave of infection from July to December 2021 in the November simulations compared to some of the June scenarios - matching the reported wave due to the Delta variant.

When considering the impact of vaccinating 12–17-year-olds on the entire population (middle row, Fig. 4), this again highlights that while infections in the 12–17 age group are relatively common (unfilled bar compared to the total height), hospital admissions and deaths are far less frequent in this age group. These plots compare the counterfactual model without childhood vaccination (grey bar) with the vaccination of 12–17-year-olds at different deployment speeds (after 6th November) and with different uptakes. Compared to the June results, the impact of childhood vaccination is diminished in the November simulations, with none of the later results generating the large reductions that were projected for some of the Step 4 scenarios. For the November scenarios, vaccination of 12–16-year-olds generates a mean 8–10% reduction in infection, hospital admissions and deaths across the general population, compared to up to 45% in some of the June scenarios. This reduced impact of vaccination can be attributed to multiple factors: the difference in the pattern and scale of infection between July and December 2021, the higher vaccine efficacy assumptions in November such that the majority of the older population have greater protection, and the slower roll-out schedule compared to earlier estimates.

### November predictions, 5–11-year-olds

**Fig. 5:**
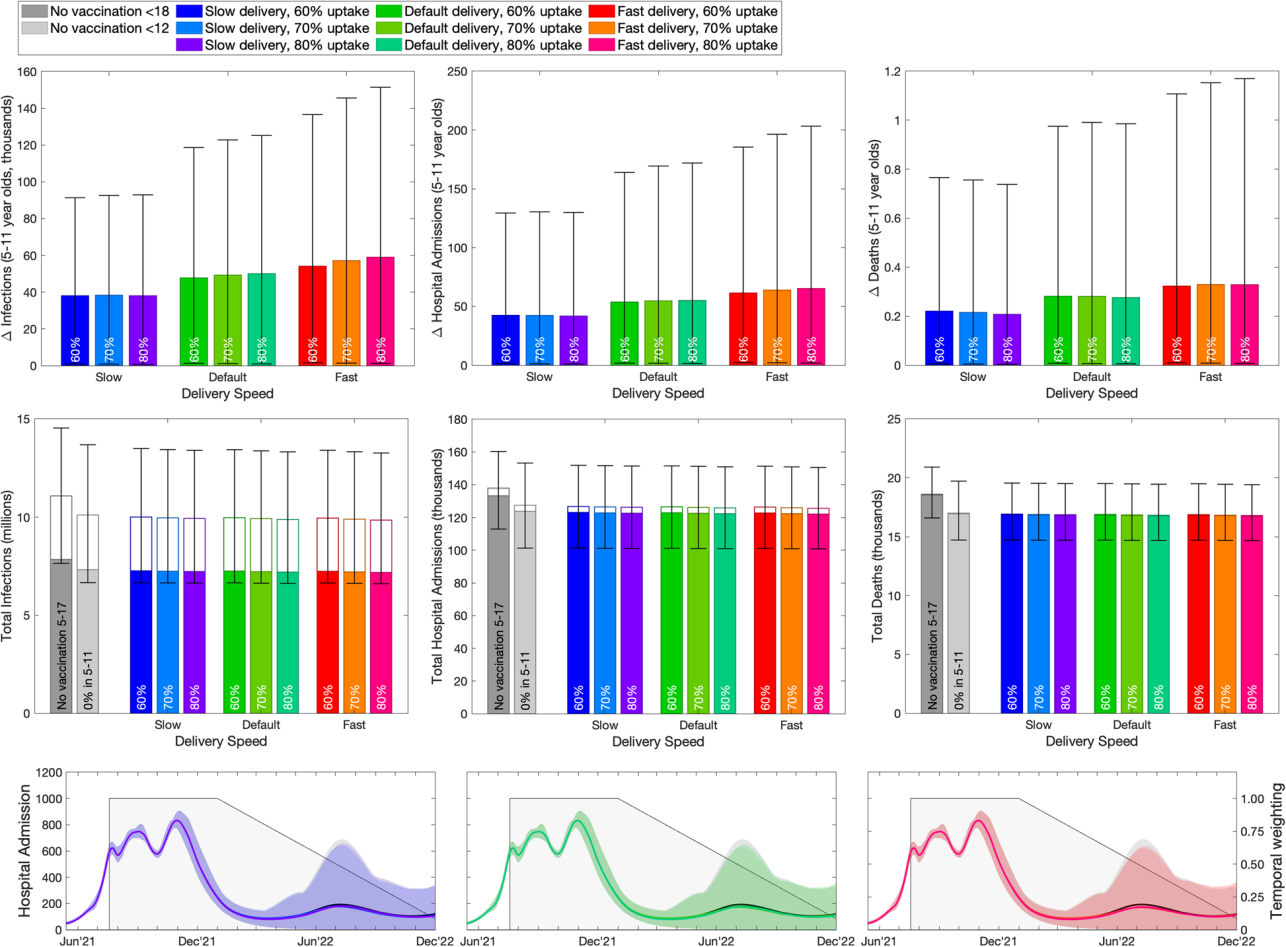
Impact of vaccination of 5–11-year-olds in England, calculated in November 2021. Top row: reduction in infections, hospital admissions and deaths in 5–11-year-olds due to vaccination in this age group. Middle row: total number of infections, hospital admissions and deaths in the entire population (total bar) and 5–17-year-olds (open bar). Lower row: number of projected hospital admissions over time (lines and ribbons; black for vaccinating those aged 12 and above but not 5–11-year-olds, colours corresponding to the bars in the above rows), and the assumed time discounting (grey shading). In the upper two rows bars are the mean value, error bars are the 95% prediction intervals, and different colours represent different assumption about uptake in 5–17-year-olds (no vaccination, 60%, 70% and 80%) and different assumptions about vaccine delivery speed (slow - 100,000 per week, default - 140,000 per week, fast - 180,000 per week). In the lower row, the counterfactual of no vaccination in all 5–17-year-olds (dark grey) and the vaccination of 12–17 but not 5–11 (light grey) are both shown

Switching attention to the vaccination of younger children aged 5–11 years (Fig. 5), it is assumed that their vaccination begins in March 2022, by which time the majority of older children (12–17) will have already been offered two doses. This later start date, coupled with the temporal weighting applied to all sums, means that there is less time (and less weight at each time point) for the vaccine to register an impact; the programme weighting has been reduced to 81 days. Therefore, while it makes sense to compare different scenarios for vaccination of 5–11-year-olds, it is difficult to compare between the two childhood programs (12–17-year-olds vs 5–11-year-olds) due to the different time-frames involved—earlier vaccination would inevitably generate greater impacts. In addition, the 5–11-year-old cohort comprises approximately 4.8 million children; hence, vaccination is likely to take longer than the earlier vaccination of 12–15 and 16–17-year-olds which was performed as two separate cohorts.

Considering the impact of vaccinating 5–11-year-olds on infection and disease in the 5–11-year-old age group (top panels, Fig. 5) delivery speed (blue - slow, green - default, red - fast) has a pronounced impact as the entire delivery programme is now being simulated. The impact of vaccine uptake is most pronounced when the roll-out is fast, as this achieves the maximum coverage in the shortest amount of time, before these individuals become infected. However, for all these results, the lower bound of the 95% prediction intervals is close to zero, showing that in at least 2.5% of the projections the vaccination of 5–11-year-olds is relatively ineffective as there is projected to be a substantial wave of infection in this age group before vaccine delivery starts. In general, the mean relative decrease in infection, hospital admissions and deaths within this younger age group is between 1 and 3%.

Again considering the impact on the entire population of vaccinating this youngest age group (bottom row, Fig. 5) highlights that vaccination of 5–11-year-olds has a minimal effect on the rest of population, partly due to the late start of the vaccine campaign meaning older individuals are already protected by immunisation or infection.

## Discussion

The SARS-CoV-2 immunisation programme for children and young persons in England has evolved in an iterative manner, which is reflected in the timing of vaccinations in the later model. From late July (JCVI advice published 19th July 2021 [67]), those aged 12–17 with specific underlying health conditions that put them at risk of serious COVID-19 were offered the vaccine. This was extended in August (JCVI advice published 4th August 2021 [68]) to a first dose to all 16–17-year-olds, a second dose 12 weeks later (JCVI advice published 15th November 2021 [69]) and a booster dose after 3 months (JCVI advice published 22nd December 2021 [70]). Those aged 12–15 (and not in a high-risk category) were offered their first doses through schools from late September (JCVI advice published 3rd September 2021 [71]), with a second dose 12 weeks later (JCVI advice published 29th November 2021 [72]). Finally, at-risk 5–11-year-olds with specific underlying health conditions that put them at risk of serious COVID-19 were offered the vaccine from late December 2021 (JCVI advice published 22nd December 2021 [70]).

As can be seen from the decisions for England, there are considerable advantages in (initially) targeting vaccination to the most vulnerable [8, 73]—applying equally well to both adults and children. Available data suggests that clinically vulnerable children comprise around half of hospital admissions and three quarters of deaths in this age group [12, 74], so protecting these individuals first with early targeted vaccination provides the greatest immediate impact. Due to a lack of available data, vulnerable groups have not been explicitly captured within our model; instead the risks (as captured by the infection:hospital ratio or infection:mortality ratio) are realised as an average over all individuals within each 5-year age group. As such our results pertain to the advantages of vaccinating entire age cohorts; once the vulnerable have been offered the vaccine, the benefit of vaccinating the non-vulnerable is likely to be significantly reduced. Calculating the size of this reduction is not simply a matter of scaling down hospital admissions and deaths, but also needs to account for the indirect impact of vaccination (protection due to the reduction of infection) in this target age group.

This paper has focused on the epidemiological impacts of vaccinating 5–17-year-olds; calculating the reduction in infection, hospital admissions and deaths over a given time period. Using the November projections, we would estimate a reduction of approximately 280 thousand infections, 610 hospital admissions and 10 deaths in the 12–17-year-old age group over the time-period studied, if at least 60% of this 3.8 million cohort were vaccinated (equating to a drop of around 25% compared to the counterfactual non-vaccination scenario); and an additional reduction of 40–60 thousand infections and 40–60 hospital admissions in the 5–11-year-old age group when vaccinating this younger age (equating to a drop of around 1–3%). In part the lower numbers from the vaccination of 5–11-year-olds comes from the lower programme weighting: 81 days compared to 267 days—due to the later impacts of the programme.

When considering the implementation of any new vaccine program it is important to consider the potential risks and costs alongside the expected benefits. In England, it is usually common to formulate a cost-benefit analysis in terms of financial costs of the vaccine against the benefit in terms of QALYs (Quality Adjusted Life Years) gained [75, 76] – although this approach has not been used when considering vaccination against SARS-CoV-2. In addition, there is now evidence that the general public expect the benefits of immunisation to substantially outweigh any health risk associated with adverse events of vaccination [77]. In this context, there were considerable early concerns about the risks of myocarditis and pericarditis following vaccination in younger individuals [78–81], and the potential for such adverse effects to disrupt the public’s confidence in the entire vaccination programme. However, there are clearly benefits associated with vaccination of 5–17-year-olds; while the saving in terms of hospital admission and deaths is moderate compared to the total numbers observed so far, the benefits to the vaccine cohorts especially in terms of minimising infection and associated risks or minimising educational losses is clear.

There are several factors that could be introduced into the modelling framework to increase its realism. As mentioned above, being able to separate the younger population into vulnerable and non-vulnerable individuals would mean that the models would more closely replicate the pattern of vaccine roll-out deployed in England. While the model generates the number of infections and hospital admissions due to COVID-19, it does not attempt to quantify long-COVID – in part due to a lack of clinical definition and hence a lack of national-scale data [82, 83]. For some scenarios, the number of individuals involved is relatively small (e.g. the reduction in deaths in 5–11-year-olds due to vaccinating this age group) in which case it may be beneficial to adopt a stochastic approach such that the numbers predicted are all integers. Finally, the models do not include the Omicron variant; data on this new variant are still relatively sparse prohibiting the ability to make robust long-term projections. The substantial Omicron wave in early 2022 would likely increase the benefits of vaccinating 12–17-year-olds due to the additional protection conferred against this wave; however if would likely reduce the benefits of vaccinating 5–11-year-olds as they are unlikely to be vaccinated before any peak of the Omicron wave. The lower vaccine efficacy and lower severity reported for the Omicron variant could also reduce the benefits of childhood vaccination—more data is needed before detailed simulations can be performed.

## Conclusions

We have shown that vaccination of 12–17-year-olds, and to a lesser extent the vaccination of 5–11-year-olds, reduces infection, hospital admissions and deaths. These reductions were projected to be higher when modelling the dynamics in June compared to November 2021, partly due to an assumption of more rapid vaccine delivery and partly due to the unexpected scale of the third wave from July to December 2021. The projections from November 2021 suggest the vaccination of 12–17-year-olds will generate a saving of around 600 (PI 280–1280) hospital admissions and a reduction of around 280,000 (PI 200,000–380,000) infections in this age group; without vaccination we project 2200 (PI 1300–4000) hospital admissions and 1.3 million (PI 1.0–1.6 million) infections. The decision to offer vaccination to these younger individuals needed to balance the benefits to both the vaccinated age group and the population in general against the risks of adverse events and the potential disruption to a range of other public health activities.

## Supplementary Information

**Additional file 1** Model Formulation. Detail of the underlying mathematical framework that defines the transmission model. We break the model desciption into multiple sections that combine to generate a picture of SARS-CoV-2 transmission in the UK.

**Additional file 2** June Projections with shorter time window. Figure showing the original assessment of vaccinating 12-17 year olds, shared with JCVI in June 2020, with used a much shorter and more abrupt time window - weighting all values between 19th July and 31st December 2021 equally.

## Data Availability

Data on cases were obtained from the COVID-19 Hospitalisation in England Surveillance System (CHESS) data set that collects detailed data on patients infected with COVID-19. Data on COVID-19 deaths were obtained from Public Health England. These data contain confidential information, with public data deposition non-permissible for socioeconomic reasons. The CHESS data resides with the National Health Service (www.nhs.gov.uk) whilst the death data are available from Public Health England (www.phe.gov.uk). The ethics of the use of these data for these purposes was agreed by Public Health England with the Governments SPI-MO / SAGE committees. More aggregate data is freely available from the UK Coronavirus dashboard: https://coronavirus.data.gov.uk/. The MATLAB code that underpins the June and November models is available at: https://github.com/MattKeeling/Vacc_SchoolAged_Children.
