# Supplementary material for: An assessment of the vaccination of school-aged children in England against SARS-CoV-2
Source: BMC Med. 2022 May 18;20:196. doi: 10.1186/s12916-022-02379-0 (PMC9113775; doi:10.1186/s12916-022-02379-0)
Supplement: Supplementary file 2 — Additional file 2 June Projections with shorter time window. Figure showing the original assessment of vaccinating 12-17 year olds, shared with JCVI in June 2020, with used a much shorter and more abrupt time window - weighting all values between 19th July and 31st December 2021 equally. [file 12916_2022_2379_MOESM2_ESM.pdf]

Additional File 2: June Projections with shorter time window

In the original assessment of vaccinating 12-17 year olds, a much shorter and more abrupt time window was used - weighting all values between 19th July and 31st December 2021 equally (S2.1). Results for the mean number of infections (top left) and hospital admissions (top centre) saved in the 12-17 age-group and the mean number of hospital admissions (middle left) across the entire population were shared with JCVI.

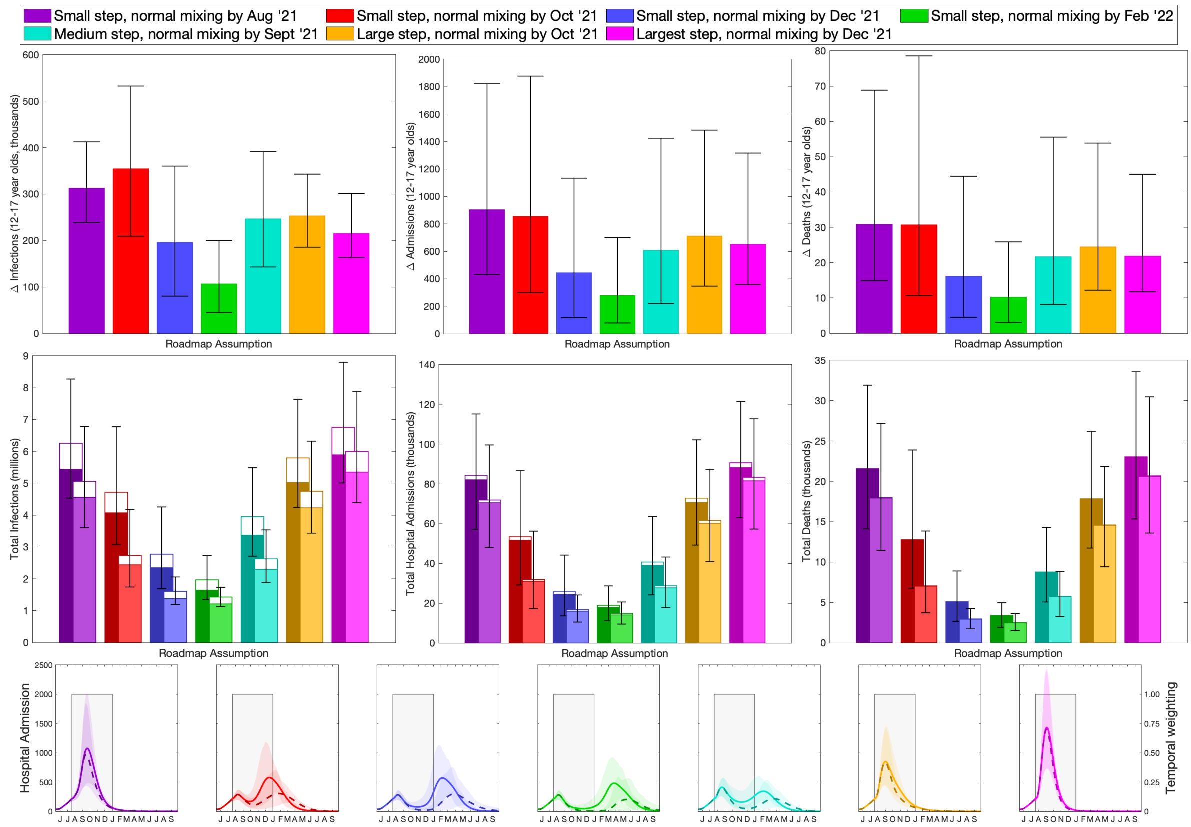

Fig. S2.1: Impact of vaccination of 12-17 year olds in England, calculated in June 2021. Top row: reduction in infections, hospital admissions and deaths in 12-17 year olds due to vaccination in this age-group. Middle row: total number of infections, hospital admissions and deaths in the entire population (total bar) and 12-17 year olds (open bar) - the darker rear bars are the projected totals without vaccination of 12-17 year olds, while the lighter front bars are the totals when 80% of 12-17 year olds receive the vaccine. Lower row: number of projected hospital admissions over time (lines and ribbons), and the assumed time discounting (grey shading). In the top two rows bars are the mean value, error bars are the 95% prediction intervals. The different colours represent different assumptions about changes in social mixing patterns after Step 4 [56]; this is modelled as a step-change on 19th July 2021, followed by a gradual return to pre-pandemic mixing over different time scales. This is the equivalent of Figure 3 in the main text, but with quantities calculated over a shorter time-window.
